# Just ethnic matching? Racial and ethnic minority students and culturally appropriate mental health provision at British universities

**DOI:** 10.1080/17482631.2022.2117444

**Published:** 2022-09-01

**Authors:** Funmi-Victoria Olaniyan, Graeme Hayes

**Affiliations:** Department of Sociology and Policy, Aston University, Birmingham, UK

**Keywords:** University students, racial microaggressions, mental health, ethnic matching, person-specific approaches

## Abstract

**Purpose:**

The need for “culturally appropriate” support for racial and ethnic minority (REM) students has prompted several British universities to embrace targeted interventions such as “ethnic matching” to encourage professional help-seeking on campus (i.e., pairing REM students with ethnically similar practitioners). There remains, however, little clarity on what culturally appropriate support entails. This study explores how REM students define culturally appropriate support and the approaches they view to be effective in promoting help-seeking.

**Methods:**

Semi-structured interviews were conducted with 48 REM students in two British universities. Data analysis was guided by principles of constructivist grounded theory and reflexive thematic analysis.

**Results:**

REM students discuss three ways universities can provide culturally appropriate support; via ethnic matching; a broader cultural appropriateness; or a person-specific service. For these students, a service narrowly focusing on race/ethnicity has the potential to remove rather than enhance accountability and engagement within mental health service provision, and not adequately valorize the experience of the student as both individual and racialized.

**Conclusion:**

A protocol-driven and instrumental understanding of “culturally appropriate” support may serve to reduce REM student willingness to seek professional help. Universities, therefore, should commit to a student-centred process, combining racial diversification and cultural recognition with a reflexive person-specific approach.

## Introduction

Racial and ethnic minority (REM) students experience a combination of systemic, social, and cultural stressors that can significantly impact their mental health (Arday, 2018; Olaniyan, 2021 2021; Sancho & Larkin, 2020). These stressors include: under-representation on degree programmes (Crozier et al., 2016); religious discrimination (Chaudry, 2021); social isolation; and racism on campus (Akel, 2019; Tate & Bagguley, 2017). Meanwhile, an increasing number of British REM students have discussed the psychological impact of racism on their mental health experiences (Arday, 2018, 2021). These factors can also impact on their ability to seek professional psychological help; for instance, research presents a mental health paradox among REM students where they are more likely to experience mental health problems yet remain less likely than the general student population to seek formal help (Arday, 2018; Sancho & Larkin, 2020; Turner et al., 2007). Reasons for this may vary; however, REM student help-seeking attitudes are likely to be influenced by mental (ill) health stigma (Soorkia et al., 2011); different worldviews regarding the causes and treatment of mental ill health (Cadge et al., 2019; Furnham et al., 2008); and fears of *double discrimination* by mental health practitioners (MHPs) or their peers (Olaniyan, 2021) . For REM students who do seek help, research suggests that they continue to experience inadequate mental health support, including being subjected to discriminatory and stereotypical judgements perpetuated by MHPs (Arday, 2018, 2021; Olaniyan, 2021).

In response, programmes such as Universities UK’s Stepchange framework (De Pury & Dicks, 2020) and the University Mental Health Charter (Hughes & Spanner, 2019) have been established to improve student mental health outcomes. These programmes call for universities to take a “whole university approach”, as “isolated interventions are inadequate to address the multifactorial challenge of multiple mental health determinants and consequences” (Hughes & Spanner, 2019, p. 10). However, a number of researchers (Macaskill, 2013; 2018; Brown, 2018), along with higher education institutions (HEIs) themselves, now recognize a need to take further action, and advocate culturally “sensitive” or “appropriate” support on university campuses. Most prominently, these feature one recognized form of culturally appropriate support, ethnic matching. The expectation is that these services will be better able to engage with the nuanced and often racialized experiences of REM students, and in turn ensure better mental health outcomes (e.g., Turner et al., 2007; Priestley et al., 2022; Sancho & Larkin, 2020). Arday finds that REM students at British universities are consistently “on the periphery of healthcare services”, leaving them isolated in the face of mental illness. Furthermore, and particularly where universities are themselves a dominant white space, campus mental health services are “often unresponsive to the specific and unique needs of ethnic minority groups”; as a result, Arday advocates the “development, commissioning, and delivery of effective and culturally applicable mental health services while additionally improving access to these services”. Included in these recommendations is greater diversification of MHPs, to create “ethnic concordance between service users and providers” (Arday, 2018, pp. 215,216).

So far, there is little evidence concerning the uptake or reception of these targeted interventions by REM students. This is important, because whilst ethnic concordance (or ethnic matching) itself appears a clear and productive way forward, the provision of culturally appropriate support may entail more than a simple checklist approach to pairing on racial and ethnic grounds. Indeed, the help-seeking decisions and preferences of REM students are likely to be both complex and highly context-dependent, shaped by multiple sociocultural and systemic factors beyond race/ethnicity. Questions regarding the meaning of culturally appropriate support and the value or effectiveness of matching approaches have long been raised in the North American mental health literature, amongst both the general population (e.g., Helms & Carter, 1991) and REM students (e.g., Swift et al., 2015). Some of this research suggests that while targeted approaches have the potential to improve REM mental health outcomes, they do not always do so; equally, there is little solid evidence that approaches such as ethnic matching provide better treatment or outcomes for REM individuals (e.g., Cabral & Smith, 2011; Knipscheer & Kleber, 2004; Maramba & Nagayama Hall, 2002). Some (e.g., K.N. Anderson et al., 2019; Karlsson, 2005; Vasquez, 2007) argue that it may be within-group variables—such as cultural attitudes, ethnic identity, social class, and the opportunity to form a “therapeutic alliance” with the MHP—that better determine the outcomes of treatment. Curtis et al. (2019) further adds that there is a possibility that REM individuals may be more interested in “cultural safety” rather than “culturally appropriate” interventions. They argue that truly “appropriate” care can be provided by any MHP regardless of their race/ethnicity, provided that they are aware of the difference between themselves and the REM client; consider power relationships; implement reflective practice; place the client’s needs at the centre of their interactions; and allow the client to determine whether their interaction with the MHP is safe and “culturally appropriate”.

These findings therefore raise a series of questions: when REM students request culturally appropriate support, do they specifically mean “ethnically matched” support? Can culturally appropriate support only be offered by REM MHPs, or can any MHP, regardless of their ethnicity, offer appropriate support provided they are sensitive to the cultural background and experiences of the student? (Batchelor et al., 2020; Mead & Bower, 2000). How do REM students view their university support services and what changes, if any, do they believe must occur to ensure these services become culturally appropriate? Here,

we seek to address these questions through discussion of the lived mental health experiences of 48 REM students in two UK HEIs. Through in-depth, semi-structured interviews, we explore how these students define a culturally appropriate service, and engage with their articulations of their needs and preferences. We find that the mental health seeking priorities of these students most often *include but go beyond* matching approaches, and that some targeted interventions may not work for all students, particularly if they are constructed to satisfy organizational targets rather than the experience of students from racialized minorities themselves. We argue as a result that a recognition of racial difference and specific culturally structured needs must be reflexively combined with person-centric approach which recognizes the student as both racialized and individual.

## Racial microaggressions, racial battle fatigue, and culturally appropriate support

Over the last fifteen years or so, research on the relationships between racism and health outcomes has increasingly focused on racial microaggressions. Initially identified by Pierce (1970), understanding of the significance of racial microaggressions was catalysed by the work of Sue, and particularly Sue et al’s landmark 2007 article, which identified a shift in the character of racism in North American settings from blatant and overt to much more subtle, everyday, and insidious expression. For Sue et al, racial microaggressions can be taxonomized into the three categories of microassaults, microinsults, and microinvalidations, and are the “brief and commonplace daily verbal, behavioural, and environmental indignities, whether intentional or unintentional, that communicate hostile, derogatory, or negative racial slights and insults to the target person or group” (D.W. Sue et al., 2007, p. 273). Racial microaggressions are the expression of what they identify as aversive racism, or the implicit and unconscious discriminations routinely practised by liberal citizens holding formally egalitarian value sets. Aversive racism is thus manifested as a “conflict between individuals” denial of personal prejudice and their actual underlying unconscious negative feelings and beliefs about people of colour’ (Constantine & Sue, 2007, p. 143).

Microaggressions work in tandem with macrostructural discriminations and exclusions; indeed, for Smith, David and Stanton (2020:87), the micro is less a question of the character of the racial aggression and more of its locus, defining microaggressions as “direct individual-level attacks on a racially marginalized person of color” (this apparent pivot from nature to scale may in fact help answer the question posed by G. Wong et al. (2014) as to why the decidedly non-subtle category of microassaults might be contained within Sue’s taxonomy). As Domínguez and Embrick (2020) emphasize, it is the cumulative effect of these aggressions, as expressions of everyday racial violence, that are particularly important; microaggressions set a causal reaction in train, acting as stressors mediating the effects of racial minority status on an individual’s psychological, physiological, and behavioural integrity, and creating negative impacts on their wellbeing, self-regard, and bodily health (G. Wong et al., 2014, pp. 193–4). Indeed, Smith and collaborators categorize the cumulative effect of the multiple psychosocial and physiological impacts of racial microaggressions as *racial battle fatigue*, or RBF (Smith 2004, Franklin, Smith and Hung 2014, Smith et al., 2016). Intervening at both the individual and group level, RBF is caused by the “constant redirection of energy needed […] to deal with race-related stress”, and is particularly prevalent in “hyper-toxic environments” (Smith et al., 2020, pp. 87, 85). Given the cultural logics at play, much of the research into racial microaggressions and the production of RBF has focused on the “hostile campus racial climates” of historically white universities and colleges in the USA (Woods et al 2021, G. Wong et al., 2014). Nonetheless, as Arday notes, the remit of RBF has now been “expanded within racial discrimination vernacular to describe the negative and racially charged experiences of [REM] people within and beyond the USA” (Arday, 2022, p. 86).

Sue’s initial response to racial microaggressions was to call for “the invisible to be made visible” (Sue, 2004), proposing toolkits and training for white mental health professionals and supervisors (Constantine & Sue, 2007). This focus on identifying and addressing unconscious bias in white MHPs chimes with wider calls for the provision of culturally appropriate services (Bhui & Sashidharan, 2003; Dreeben, 2001; Herman et al., 2007; Memon et al., 2016; Zane & Ku, 2014). These calls have often been influenced by research that indicates that the cultural competence of a MHP or service impacts upon service satisfaction for REM groups. For example, C.E. Thompson et al. (1994) found that African American women engaged in greater depth of self-disclosure and were more willing to attend therapy when their counsellor attended to their cultural context. Similarly, Keating and Robertson (2004) found that people from a Black British background who had prior negative experiences with mental health services believed that their treatment and diagnosis would (or might) have been different had they been in contact with a member of staff who understood their experiences as a Black person. In response to such findings, several activities have been proposed, involving more REM groups in patient, public involvement programmes, MHPs becoming better equipped to handle cultural and faith beliefs or developing understanding around their recognition of symptoms and how these are expressed in different ethnic groups (Bignall et al., 2019).

Yet, despite widespread endorsement of these proposals by researchers, public health professionals and REM communities themselves, social and racial gaps still remain in mental health outcomes (Baskin et al., 2021). There are many potential reasons for this, and perhaps one of the most obvious obstacles to improving mental health outcomes through the provision of culturally appropriate services is the lack of agreement on a definition of “culturally appropriate” as well as a lack of adequate descriptors for this type of support. Increasingly, culturally appropriate support is translated as an ethnic matching approach, as observed in a number of British HEIs. Some studies argue that matching has increased the likelihood that REM clients trust their counsellors (E.C. Wong et al., 2003; Meyer et al., 2011; V.L.S. Thompson et al., 2004), or produced better treatment outcomes and retention (Dana et al., 2001; S. Sue et al., 1991). Testing the effect of racial matching on therapist credibility, Meyer et al. (2011) found that racially matched individuals rated the therapist as more credible; crucially, this rating was mediated by the perception that the therapist shared similar life experiences to the client. British REM groups tend to express wishes to see a service provider from the same background as themselves for similar reasons of experiential similarity (Bhui & Sashidharan, 2003), whilst those who have previously experienced racial discrimination in healthcare tend also to prefer matching approaches (Memon et al., 2016).

## Cultural competence, ethnic matching, and beyond

However, another body of research shows that emphasis on this type of matching may not always influence or improve client outcomes, nor is it always the client’s preference (e.g., Maramba & Nagayama Hall, 2002; Knipscheer & Kleber, 2004; Cabral & Smith, 2011). For example, Swift et al. (2015) found that REM students valued working with an MHP with cultural training and experience of the systemic issues REM individuals face much higher than being matched to an MHP based on their race/ethnicity. Furthermore, meta-analysis of racial matching did not find worse outcomes for unmatched Asian American clients and counsellors (Meyer et al., 2011). Other meta-analytic reviews indicate that ethnic match only played a minor role in therapy retention or outcomes, and it did not have lasting effects on therapy after the initial sessions (Cabral & Smith, 2011; Maramba & Nagayama Hall, 2002). In many British studies, participants are often asked what they think would make services more accessible, but their responses are not empirically tested. Only a small number of studies in this area have focused on factors which have been personally experienced by participants as making services easier to access (see, Anthony, 2015).

For Muaygil (2018, p. 17), consistently overlooking limitations like these has caused understanding of culturally appropriate support to lose nuance, and has “resulted in pre-set, superficial ‘check lists’ that appear to generally, and inaccurately, outline certain characteristics and behaviours of various cultures”. He argues that culturally appropriate interventions can be ethically problematic because, in a bid to appear culturally competent, services and healthcare professionals begin to adopt untested practices and behaviours for the presumed “cultural good” of others. Through these behaviours and practices, they gather “sweeping, inflexible generalisations and develop hasty, unrestrained willingness to accept” and cater to cultural differences amongst their clients, without further consideration of how certain targeted interventions may cause harm or not be appropriate for all REM groups.

Meanwhile, others have commented on the potential harms of a narrow understanding of culturally appropriate support, as it can ignore the extensive heterogeneity of REM groups and focus solely on a limited number of categories or variables. In turn, this may provide limited information, and risk glossing or masking important within-group variations (Zane & Ku, 2014), such as the prevalence of mental illness. Whilst rates of depression are typically much higher amongst REM than White communities (Memon et al., 2016), evidence also suggests that African Caribbean people are three to five times more likely than any other REM group to be diagnosed and admitted to hospital for schizophrenia (Bignall et al., 2019; Pinto et al., 2008). There are also differences in patterns of help-seeking. Furnham et al. (2008) found that although British Pakistani students tended to use biological and genetic causal explanations for mental health problems, they were still more likely to seek help through their faith or religious beliefs than formal psychological interventions. Moreover, Moller et al found in their qualitative study of attitudes towards mental health seeking amongst young second-generation British South Asian women that this group held a series of negative generalizations about counselling, counsellors and psychological distress. This included widespread beliefs that White counsellors were “ignorant” and South Asian counsellors “gossipy”. As a result, they argue, the key to removing barriers to mental health-seeking lies in addressing these stereotypes directly (in both community-directed general information provision and during the counselling process itself). For White counsellors, the priority lies in “the need to address assumptions of cultural ignorance, which may actually involve acknowledging cultural ignorance” (Moller et al., 2016, p. 208).

As a result, the difficulty in achieving culturally appropriate support may not stem from the absence or presence of appropriate interventions, but from our limited understanding of the provider–patient dyad in its context. Indeed, it is the situation of MHP-student interactions in their specific environment that may ultimately determine whether or not a service is (and is understood to be) culturally appropriate (Herman et al., 2007). Thus although it is essential that MHPs are technically competent, a simplistic emphasis on matching risks erasing the client’s individual cultural identity and promoting reductionist understandings based on cultural misconceptions, whilst increasing the likelihood of homogenizing REM individuals into a collective “they” (Curtis et al., 2019; Kim, 2018). As Greenberg et al note in their review of the literature, “the impact of racial/ethnic similarity and difference in the therapeutic relationship is complex; race and ethnicity intersect and interact with other factors related to the worker and client in shaping how similarity and difference affect the therapeutic process” (Greenberg et al., 2018, p. 60). Indeed, even where a more “culturally competent” approach is taken instead of an ethnic matching approach (i.e., where White MHPs are encouraged to focus on and engage specifically with the cultural context of the client’s illness) there can still be disadvantages.

Some researchers now therefore advocate for a move towards more person-focused support, or the use of alternative concepts through metaphors such as cultural “safety” or “humility” (Curtis et al., 2019; Muaygil, 2018; Papps & Ramsden, 1996). Although there is overlap between these concepts and culturally appropriate support, the metaphors have different connotations, and they emphasize distinct approaches to social and cultural dimensions of support. Thus cultural safety does not emphasize developing “competence” through acquiring broad cultural knowledge, but instead emphasizes recognizing the social, historical, political, and economic circumstances that create inequalities in health and the clinical encounter (Kirmayer, 2012; L.M. Anderson et al., 2003). Cultural humility, on the other hand, is focused on the adoption by the counsellor of a “multicultural orientation” within the therapeutic dyad, enabling the counsellor to foreground and recognize difference within an interpersonal stance that is

“other-oriented (or open to the other) in relation to aspects of cultural identity that are most important to the client” (Hook et al., 2013, p. 354; see, also, 2016). These approaches encourage MHPs to (a) identify and display behaviours, skills, and attitudes and health care environment characteristics that REM individuals desire from their providers; and (b) provide opportunities for REM individuals to express these desires through ongoing feedback to MHPs about how well these desires are being met. In other words, it extends beyond an emphasis on a display of cultural competence or the provision of an ethnically matched service to a dynamic and interactional emphasis on ascertaining what clients want, need, perceive, and feel in the process of receiving care (Herman et al., 2007).

## Towards culturally appropriate support in UK universities

The context of British student support services is therefore interesting. As we have underlined elsewhere, research on the relationships between the British university environment, racial microaggressions, REM student mental health, and the counselling interaction is an underdeveloped yet growing field; Stoll et al. (2022) identify a corpus of twelve articles addressing mental health and mental well-being experiences among Black students at UK universities published between 2010 and 2020. Clearly, this literature emerges from a different cultural context from the USA, which is structured by the legacy of plantation slavery and Jim Crow; nonetheless, work by Arday (2018), Akel (2019), Bunce et al (2019) and others identify the commonplace nature of racial abuse, both overt and implicit, experienced by racialized minority students within the inequitable environments of British universities. Synthesizing, Stoll et al identify an experience common to Black students of being subjected to racism and racial stereotyping in the classroom, through teaching materials and staff-student and student-student interactions; and for Black women, in addition, being subjected to the overpolicing of their academic knowledge by teaching staff (Stoll et al., 2022, p. 6). Five of their corpus of twelve studies discussed how “institutional racism, discrimination and hegemonic white privilege made universities toxic spaces for Black students, which affected their mental health and well-being” (Stoll et al., 2022, p. 7). The lead author’s own work, completed for the study of which this paper is a part, has identified REM students as typically experiencing campus climates as hostile and characterized by everyday racial microaggressions, multiple types of othering, and tokenist institutional approaches to social and racial diversity (Olaniyan, 2021) . As Williams et al. (2021) note, tokenism is of course itself a form of racial microaggression.

At the same time, spurred by official reports, some UK universities have latterly attempted to reform the provision of student counselling services, with the goal of making them more “culturally appropriate”.

An internal review conducted by The University of Edinburgh (2019) identified a gap between the awareness and racial literacy of university staff and the lived experiences of both UK-domiciled and international black and minority ethnic students, and found that REM students experience barriers accessing support services at the University. The University now recommends that their support services consider using positive action to diversify their staffing and so provide an “ethnically matched” service. There are multiple signs that this specific matching approach is gaining currency across UK HEIs.^1^ For reasons apparent from our discussion of the literature, above, we suggest that these attempts should be treated as positive, yet also potentially problematic—not least because there is as yet no universal definition for culturally appropriate support; nor is there robust research addressing what students define to be culturally appropriate support, or set guidelines on how universities should approach the provision of such support.

In particular, we think it especially important that UK HEIs do not limit themselves to an underlying assumption of a “finality” to understanding issues of race/ethnicity and mental health; and that they do not adopt the view that “cultural appropriateness” can be simply learnt and actioned through an instrumentalised checklist. Above all, given the performative and rent-seeking nature of UK HEIs where racism is concerned (Bhopal & Pitkin, 2020), we fear the embedding of tokenistic approaches over the development of reflexive and structural responses. In previous work, we have seen that REM students are wary of diversity approaches because they have already negatively experienced protocol-driven encounters with university and external mental health services (Olaniyan, 2021), , and are wary of their replication within professional services encounters. Indeed, we argue that these issues must be consistently grappled with (Dogra et al., 2007). That is, HEIs should welcome the idea that appropriate support may not necessarily mean the development of separate interventions (Brown, 2018) for REM students; rather, appropriate support may be achieved through MHPs being aware of difference, considering power relationships, and by allowing the “patient” to determine whether their interaction with a MHP is or is not culturally appropriate. This is imperative, especially in a time where despite various commitments to, and initiatives for improving REM student mental health, British REM students with mental health problems are still being “failed throughout the student cycle” (Office for Students, 2019).

## Research design and methods

Drawing on a more extensive study (Olaniyan, 2021) of the influence of different campus environments on REM student mental health and help-seeking attitudes, here we adopt a qualitative approach to determine how REM students at two neighbouring British HEIs conceptualize culturally appropriate support. The two HEIs have specific differences and similarities: whilst both are selective, and (by some yardsticks) high performing, they have marked differences in the social and ethnic diversity of the student body. At both HEIs, there is nonetheless a structural similarity to their approach to campus mental health service delivery, with neither explicitly adopting a culturally specific or ethnic matching approach.^2^

Our sample is composed of 48 UK-domiciled REM students. This is somewhat larger than is typical for qualitative work on racial microaggressions; in their early review of the blossoming psychology literature, Wong et al found that analyses gathering qualitative data (n = 38/73) had sample sizes ranging from five to 97, with an average sample size of around nineteen (G. Wong et al., 2014, p. 195). To recruit our sample, we deployed a combination of convenience and purposive sampling methods; we chose these methods due to their applicability to this type of approach (Smith et al., 2016, p. 1196), and widely accepted effectiveness in recruiting potentially *hard-to-reach* research participants (Vickers et al., 2012). The lead author approached students at the meetings of societies and events catering to REM students (Afro- Caribbean Society, African Caribbean Medical Society, and Multi-faith chaplaincy events); and advertised the study by placing physical recruitment posters on each campus and on social media via student forums. As we aimed to explore the lived experiences and perspectives of students across a range of backgrounds, mental health statuses, and help-seeking histories, we did not (knowingly) include or exclude students who would be expected to seek or privilege ethnic matching; this includes those who were currently undergoing treatment for mental ill health (on campus or elsewhere) or those who previously had been. Our final sample is biased towards women (n = 30; 62.5%), and students from a Black British (n = 32) and South Asian-British background (n = 16). Thirteen (27%) participants had a current professional diagnosis of depression (n = 8), anxiety (n = 4) or bipolar disorder (n = 1); 21 (44%) had self-defined mental health problems which they commonly described as symptoms of depression or anxiety; 25 (52%) had prior help-seeking experiences either through campus services (n = 10), off campus services (n = 10), or community/faith-based services (n = 5). At the time of interview, three participants were currently engaged with their university’s support services and three were managing their own symptoms. The full demographic characteristics of the participants are presented in Supplementary File 1.

### Data collection and analysis

The lead author conducted semi-structured interviews with the participants between December 2018 and November 2019; 22 face-to-face in a private room on campus, and 26 by telephone. The interviews lasted between 23 and 113 minutes, were audio recorded, transcribed, and transferred into NVivo-12 qualitative analysis software for data management and analysis. The lead author asked participants general questions about their: understanding of mental health and wellbeing; experiences of mental ill health and help-seeking; experiences of life on campus; knowledge and perceptions of their university’s existing support services. They also asked specific questions exploring participants’ preferred characteristics in a mental health provider/service, and recommendations for their university’s existing services.

To analyse the data, we were guided by principles of constructivist grounded theory (CGT) and reflexive thematic analysis. The lead author transcribed and analysed each interview transcript consecutively starting with a process of initial coding, a core feature of CGT (Charmaz, 2014). Initial coding involves understanding and interpreting sections of data and labelling it with a word or phrase, preferably stemming from the participants’ own words (Charmaz, 2014; Corbin & Strauss, 2014). During initial coding, the lead identified connections between interview data and used these connections to categorize the raw data in a more analytically sound format. Throughout this stage, it was essential to remain open to exploring any theoretical direction that emerges from the data (Charmaz, 2014; Corbin & Strauss, 2014). Following initial coding, both authors began a second level of data analysis (or “focused” coding) where we compared and combined multiple codes with characteristic similarity to create broad themes. We then refined the broad themes by patterns, context, and meaning. At this stage, the data analysis process was transformed from a descriptive to a conceptual level (Charmaz, 2014; Clarke & Braun, 2017; Nowell et al., 2017).

### Researcher positionality

As Simpson and Mayr (2010, p. 11) underline, all forms of institutional discourse are characterized by asymmetrical speaking rights and obligations. Moreover, authority is relational, and expressed through the social positioning of the interviewer, notably by where they stand in relation to the “other” (Byrne, 2018). Understanding and mitigating for the effects power in qualitative interviewing is therefore crucial. Much of the discussion in the methods literature of mitigation strategies emphasizes matching approaches; Merriam et al, for example, stress the “more one is like the participants in terms of culture, gender, race, socio-economic class and so on, the more it is assumed that access will be granted, meanings shared, and validity of findings assured” (Merriam et al., 2001, p. 406).

Given the focus of this study itself concerns the appropriateness of matching, this requires some unpacking. In our case, the lead author identifies as a British Nigerian woman, and therefore an REM researcher; in the terms of this study, therefore, an insider researcher whose social positionality should enable access to a hard to find (or rather, “easy to ignore”, NBCWN, 2008) constituency; and, once access is secured, enhance the interview process through empathy, mutual rapport, and trust (Kanuha, 2000; Dwyer & Buckle, 2009). Indeed, as a Black British African woman, the lead author found that interviewees considered her a cultural insider, and as such was privileged to be trusted by most respondents (especially the Black African/Caribbean students), to the extent that they were sometimes willing to share highly sensitive information. More importantly, participants often felt free enough to share their views and beliefs regardless of how controversial those views might have been. The lead author found that “silent understandings, culture-bound phrases that did not need interpretation, and non-verbalised answers conveyed with hand gestures and facial expressions” (Johnson-Bailey, 1999, p. 669) were a recurring feature of the interviews. Through these, we gained rich, in-depth, and detailed data.

The lead author being a Black PhD student might therefore be viewed as advantageous, not least because she too has experienced microaggressions and exclusions with varying effects on her health and wellbeing. Yet this positionality also brings complications. As Dwyer and Buckle (2009, p. 58) note, assumptions of similarity can also create misapprehensions of individual experience, with the potential production of an interview led by the interviewer’s, and not the participant’s, experience. This is, for Pitman (2002, p. 285), the “illusion of sameness”; rather than a simple social or racial matching process, the insider researcher particularly requires a critical and reflexive position through their own knowledge production process, enabling the generation of what Dwyer and Buckle term the “space between”. For us, this meant that the lead author used multiple reflexive techniques, including self-interviewing and “stream of consciousness writing” (Van Heugten, 2004, p. 207); these techniques helped distinguish her own thoughts and beliefs from those of the participants. In addition, both authors would regularly discuss the findings to ensure an accurate presentation of participants’ experiences as best as possible, with minimal filtering. As noted, given the subject of this study, our attempts to generate the “space between” was central not just to data gathering, but to the direction of our data analysis; again, this explains our choice of grounded and reflexive analytical frameworks.

### Ethical considerations and potential design limitations

We received ethical approval for this study from our host institution’s Research Ethics Committee. We obtained written informed consent from all participants and provided them with the opportunity to withdraw their consent at any time during the research process, as well as details on where to obtain support if interview topics caused distress. All interviewees are anonymized. Participants received no compensation for their participation.

The study has potential limitations. It presents the perspectives of REM students at two HEIs in one city; the extent to which these findings can be generalized beyond these cases may be limited by factors not accounted for during the design of this study. Equally, many students were Black British (African or Caribbean heritage) and women. Although this study was not designed to make generalizations of the population under study or to test a hypothesis, it is important to note that our sample was not a representative sample of the British REM population. This may be due to the recruitment of many participants from similar student groups and societies, the limits of convenience sampling (i.e., recruitment of participants with similar characteristics) and the positionality of the lead author, whose social identity potentially biased participant recruitment. Our findings cannot therefore be generalized to all British REM students. Nevertheless, these risks are mitigated by the precise focus of the research design, and by the methods of data analysis outlined above.

## REM Student preferences: cultural appropriateness, ethnic matching, and personal and reflexive

In their interview responses, REM students at both HEIs shared that a lack of racial and ethnic diversity in their university’s support services workforce often made them reluctant to access the services. For some, having access to a diverse service was especially important due to past experiences with MHPs who could not engage with topics surrounding race, racism, and religion. Indeed, the vast majority of participants (n = 21/25) who had previous professional help-seeking experiences had only encountered non-REM MHPs, whether on or off campus. For other students, the need for a diverse service was due to their understanding of the nature of the mental healthcare and counselling profession. That is, they felt that White MHPs are trained in practice within a Eurocentric diagnostic framework, and are thus more likely to misinterpret the behaviours and distress of REM individuals:

I felt a complete disconnect. I didn’t feel comfortable. I didn’t feel like what I was saying was relevant to him. He was a white male. (Sharon)

I tried the GP, but they didn’t really take it too seriously. He was so shocked to see a black person. I wouldn’t go again because of that to be honest. I think it’s because mental illness is difficult for GPs to recognise in us. (Tope)

I haven’t had the same reality as a typical White student. So, they wouldn’t be able to empathise, they can acknowledge but they can’t empathise – it’s not the way they were trained. (Grace)

I wouldn’t feel comfortable referring my friends to the services on campus – I feel like that the services right now are set up to cater to the issues White people face and only White people can understand. They need more training or to recruit more ethnic minority counsellors. If I went in or my friends and started talking about our issues such as the black tax, racism and microaggressions, we would get blank stares. (Natalia)

REM students also suggested a need to diversify the advertising and marketing strategies employed by their HEI’s support services to ensure better engagement among REM students. Two participants specifically revealed that they did not view their university services as something they could access as it did not seem as it was designed to reach REM individuals. Others critiqued the paternalistic approach of the support services, and recommended an increase in collaborative initiatives with groups that already have healthy relationships with REM communities:

Because services are not necessarily advertised to people like us. It makes it hard. I feel no one advertised to me so how will I know that I can use it too and it’s not just for White people? (Bose)

A lot of services just wait for people to come to them, but they need to start engaging with people a lot more. (Ama)

They need to be more forward facing and seem like they actually care. It needs to be more than a tick box for them. (Samira)

In general, the respondents started from a position that is familiar from the literature: in other words, that campus mental health services are structured by an implicit Whiteness, that this is a significant barrier to their capacity to access these services, that as REM students they have specific experiences of racial microaggressions not shared by White students, and that significant reform of counselling services, including the racial and ethnic diversification of MHPs, is a necessary first step in dismantling these barriers. However, some respondents added a significant caveat to these recommendations. For example, one participant believed that “ethnic-matching” solutions reflected *a lack of accountability* on the part of their university, and an *unwillingness to engage* with REM students by White MHPs. Other respondents went further: they suggested that an ethnic match may itself be problematic, removing the objectivity required to form a genuine therapeutic relationship, or *closing the space between*.

For students from a South Asian background in particular, ethnic matching was consistently viewed negatively, as it created potential conflicts with and judgement from their wider community (whom the MHP may also be a member of) who may not agree with or understand seeking psychological help:

If they make it an Asian male it’s a bit patronising ‘oh wait I can’t or don’t want to handle these people get someone else […] one of their own’. (Ajay)

To be honest I think if he was similar to me […] I don’t think he would be sympathetic. I can imagine them asking ‘what have you got to be depressed about?’ (Tina)

I’ve even had a Black counsellor at [private service] who didn’t seem to get it – it was the White woman that took the time to understand how growing up where I did affects me. (Fadeke)

I could never choose that option, the next thing I’ll hear is that need more prayers, or I’ll just see him at mosque with my dad the next day talking about what I’ve told him in confidence. (Mahmood)

White people or people not from an ethnic background […] tend to be a lot more supportive. Its more awareness. It’s not like they’re [REM communities] not intelligent but they just don’t know. Sometimes the things my parents come out with in terms of mental health, I’m like ‘what?’ [laughs]. So, I can’t imagine an Asian counsellor being any different. (Abdil)

Despite some of these objections, the respondents still believed in general that having common ground with an MHP was important. However, further discussions revealed that this common ground did not always have to involve ethnic matching. For example, one participant argued that ethnic matching could not work for all REM groups due to the heterogeneity of cultures and experiences within different communities:

There would still be cultural barriers in the way. Even if they are an Asian, they may not be Somali or Muslim so they can’t understand. There’s not enough people who can understand FGM [female genital mutilation], forced marriage and even, like, the patriarchy. (Samira)

More participants suggested that ethnic matching would not work anyway, unless this form of support was coupled with behavioural or attitudinal changes across the structure of service provision:

It’s one thing having more minorities on the frontlines, but what about their managers? Do they have biases that are inherently racist? There is no way that it won’t seep down to the therapy we receive from the Black or Asian counsellors – they [service leadership] need to change their thinking. It’s not just about employing more of us. (Andre)

I don’t think I prefer it … [ethnic specific workforce] I just think their mind needs to work a certain way. (Dwayne)

As a whole, therefore, the responses reveal a complex picture. Most see ethnic matching as a necessary starting point for service provision reform. Yet there are considerable nuances. Some respondents welcome ethnic matching, but see it as a tokenistic exercise if not accompanied by structural reform. Others argue that it can be a *get out jail free card* for White MHPs, if it means delegating REM students to REM practitioners and thus absolving them of the need to reflect on their own assumptions and practices. Others, however, consider ethnic matching itself as comprising potential pitfalls, both structurally (in that similarity elides the difference necessary to developing mutual comprehension within the therapeutic dyad); and—particularly (though not exclusively) for British South Asians—because ethnic matching might exacerbate the *shaming potential* of the clinical encounter.

## Culturally focused services

Many of these ideas recurred in thinking through the need for a more culturally appropriate approach to mental health service provision. For many respondents, in an appropriately culturally focused service, the MHP, regardless of their own race/ethnicity would be able to understand and genuinely engage with the unique context in which REM individuals’ mental ill health and help-seeking attitudes stemmed from:

There’s nothing wrong with the structures in place … that’s not why people won’t go to them. But they [the MHPs] don’t think: why are the people the way they are? What are their social environments like? What culture are they from? What is their religion? (Abdil)

With us, mental health comes in layers […] a lot of counsellors just look at the condition and forget about the stuff in between that got them to this point. So, the racism, the culture, the microaggressions. (Rachel)

This perspective was especially prominent where respondents discussed the intersection between their faith beliefs and help-seeking attitudes. For many, faith represented a familiar method for dealing with their problems, and the ability of an MHP to engage in conversations around religion represented a core element of culturally appropriate support. This was captured by Samira, who when asked to rank various sources of mental health support from least to most preferred, presented the concept of a “scale of relatability”. For Samira, private prayer was the most relatable source of help, while formal help-seeking was the least relatable. Others echoed Samira’s response. For instance, one participant shared that the reason she has maintained using the services of a White MHP (off-campus) was because they both shared the same Christian faith:

I want people that know God … I want someone I can tell God spoke to me about this. I don’t want someone that will snigger. (Dapo)

As a practicing Christian […] there’s so much to think about, will I be able to talk about my beliefs? Will they minimise my experiences? (Ebele)

He’s actually a Church of England vicar, so he gets it when I talk about God and stuff. It’s easy with him. (Tina)

Here, the *capacity to engage culturally* is key. However, upon further exploration, participants typically began to highlight that culturally focused services, like an ethnically matched service, also had limited value if the service and/or MHP was unable to “engage with the individual instead of the stereotype” (Dapo), or to put it another way, “go off the vibe I give you” (Latrice). That is, whilst attempting to provide a culturally focused service, MHPs must ensure they treat REM students as unique individuals, and not (as Samira suggested) according to “what they’ve read or learned about Black people and Muslims”.

Yes, there are some nuances […] don’t […] base our treatment on that. ‘Oh, she’s Black she must have gone through this’ … see me as an individual. Go off the vibe I give you not what you expect to find. (Latrice)

I worry that if someone starts off being accepting of the culture and everything or like is even trained to, they might label me as the things I’ve been through. (Sarah)

As we can see, therefore, a culturally focused approach differs in key respects from an ethnic matching approach, in that it requires not ostensible similarity but rather a culturally sensitive capacity to empathize, or as Samira put it, “relate”. Yet even here, this is nuanced, with some respondents emphasizing that they seek a counselling encounter which foregrounds their cultural background and specific needs, but does so in terms of their individual requirements, and does not just cast them as an expression of a collective cultural grouping. Discussion then turned to how HEIs can provide culturally appropriate support without reductively focusing on the help-seeking student’s race/ethnicity or cultural background.

## A reflexive and person specific service

Towards the end of their interviews, respondents typically began to stress that despite the need for and value of targeted interventions, the MHP and/or service *could not be properly culturally appropriate until they also become person specific*. Some participants revealed that it was not the lack of ethnically matched or culturally focused services that influenced their (un)willingness to seek help; rather, it was the opportunity (or lack thereof) to encounter an MHP and/or service they could trust with their whole selves. Here, trust is not always built through an interaction defined by a priori similarity; rather, it is built where the MHP treats them “as a human being” (Dapo) and where they (the MHP) are also perceived to be a “good human” (Aleema):

Someone that treats me like a human being and not a caseload. I don’t want it to feel like that I’m talking to a mental health professional, just a friend. […] I just want someone … very welcoming, listens to me. (Deborah)

Be patient with us, don’t be judgmental and don’t stereotype us first without getting to know us. (Andre)

To be honest I just want to be able to walk in to the [service] and know that regardless of who I meet, Black or White … I will be able to speak my truth without fear of being judged, misunderstood, or not taken seriously. (Sarah)

Indeed, one respondent had stopped using the services of an REM MHP (off campus) and was currently working with a White MHP. She shared that the main reason she had chosen this non-REM MHP was because she felt the MHP was “a good listener”:

I know people prefer someone of the same race, but I don’t see how that would help. I guess I’m lucky to finally have a therapist that I can talk to. (Fadeke)

For some students, the appeal to “humanity” could be demonstrated by modifying the settings in which mental health services are provided. They suggested the use of “clinical” settings did not feel “human” (Ama), often reinforced the stigma of “having something” (Ajay), and would deter them from using a service regardless of the service approach taken:

And they should try and be chill. Like not so clinical because that could put people off. (Kate)

Just to make it more colourful [laughs] less clinical […] and like make it seem like it’s for everyone. (Grace)

A nice lounge area it doesn’t have to feel clinical. I don’t want to be viewed as a patient. (Samantha)

The appeal to humanity has two different connotations here. On one level, it is to take the counselling encounter out of the framework of the clinical environment. One the other, it is to foreground the needs of the individual and make those needs central to the encounter. Taken together, respondents strongly emphasized that university support services should commit to a more personalized approach to service delivery in which they (i) acknowledge and engage with racial difference, but (ii) move reflexively from this cultural difference to the individual at the heart of the interaction. Indeed, the second part of this equation is dependent on the first: only through the prior recognition of the racialized nature of the encounter can the encounter become person-centred. Whilst, for example, Grace in particular did not believe that REM students had ethnically or culturally specific mental health *conditions*, she also acknowledged that their REM status influenced their *engagement* with their mental health and their preferences for support. As a result, the *professional capacity to relate* with the student as a unique individual, engage with their concerns, culture, beliefs, and identity, and do so without labelling them or providing advice according to seemingly culturally specific preconceptions was viewed as most important, regardless of the service approach taken:

It [REM status] influences your identity and shapes your experiences, not the mental health condition. (Grace)

Just treat them as a human but be aware that their cultural backgrounds may influence their life and experiences. (Padma)

I think it should be personalised as well not just using what you’ve learned from a textbook it should be from my situation. Use what you know and what I’ve told you to help me. Not just a generic textbook thing. (Ama)

To foreground the mental health seeking student as an individual does not therefore pre-suppose colour-blind approaches to health provision; on the contrary, it requires the integration of cultural difference into the structural framework of the encounter, and the capacity of the MHP to move reflexively (and without prejudice, as Andre and Sarah underline) from and through this framework to the specific needs of the individual. This is not, as Ama says, “just a generic textbook thing”—in other words, a superficial or tick-box exercise in diversity reporting. Rather, the person-focused counselling encounter requires a structural engagement with racial difference and needs.

## Discussion

Attempts at addressing the mental health and help-seeking inequalities observed amongst REM students has resulted in the growing recognition of the need for “culturally appropriate” support on campus. Here, by taking an in-depth qualitative approach, we have sought to explore how REM university students themselves understand what appropriate support means; and consequently, what their recommendations for improving their university’s support services in terms of access, strategy, and delivery are.

As detailed above, respondents consistently acknowledged the lack of racial diversity to be a barrier to mental health help-seeking on campus. For some, ethnic matching was vital; at the same time, however, respondents rarely advocated this approach as either the *only* or *sufficient* solution to the problem. Indeed, many believed that a sole focus on this support would not be beneficial; and for some, mostly but not exclusively from a British South Asian background, this would be actively harmful, as an ethnically matched counsellor may lack the “space between” that a non-ethnically similar practitioner could bring. This finding is to some extent consistent with Knipscheer and Kleber’s findings (Knipscheer & Kleber, 2004) that while ethnic similarity between the practitioner and help-seeker can be beneficial, it can also serve to “trap” the practitioner and help-seeker in their own REM identities and remove the human element of therapy. Respondents also emphasized an important corollary: if employing REM MHPs is done with the goal of creating matched dyads, this can serve to maintain White MHPs in their own trap, where the latter are absolved of the need (individually and organizationally) to engage with racial difference.

Equally, this narrow approach leaves the responsibility for solving health inequalities or addressing poor help-seeking outcomes with the affected individuals or communities; especially the REM practitioners who are recruited for the purpose of delivering these “culturally appropriate” services. These observations are consistent with Curtis et al. (2019) who argue that it is not lack of awareness about “the culture of other groups” that is driving health care inequities; rather, inequities are primarily the result of unequal power relationships, static approaches to support, “othering”, biases, unexamined privilege, and institutional racism. They argue that the appropriate response does not focus on learning the cultural customs of different ethnic groups (cultural competence) or the development of separate services for these groups. Instead, service provision should strive for “cultural safety”, which seeks to provide better support through awareness of difference and power relationships, decolonizing, reflective practice, and by allowing the individual to determine whether a service is “appropriate” or not. Indeed, in this context, rather than viewing cultural appropriateness through a narrow or binary lens of ethnic or cultural matching, practitioners and support services should examine themselves, the potential impact of their own culture on interactions and the ways their attitudes and assumptions may have perpetuated the narrative of REM students as the “exotic” other.

Thus a “culturally focused” service was a necessary precondition for most respondents, where the practitioner would address and/or engage with the cultural background of the help-seeker. Yet this was accompanied by concern that practitioners would focus solely on or label them according to the role their culture or beliefs play in determining their mental health and help-seeking attitudes, rather than focusing on them as individuals with needs beyond their background related stressors. Instead, they desired a more “person-specific” approach. This perspective was consistent across all respondents, regardless of their reported mental health status, help-seeking history, or specific REM background. Here, “humanity” takes a central position, starting from but then decentring the foundational importance of cultural difference. This is understood as the capacity of the practitioner to relate to the student as a unique individual, and to be sensitive to their concerns, cultures, beliefs, and identities, but without interacting with them according to seemingly culturally specific stereotypes or preconceptions (the “textbook”). This approach is fostered through reflexive interpersonal skills (i.e., listening skills, non-judgemental nature) and can be linked to environmental factors (making consultations less clinical in appearance). The general concept of person-specific support or “patient-centred” practice is of course widely established, and has become influential in thinking about mental healthcare provision (Mead & Bower, 2000). What emerges here is that the desire for a person-centric approach is not systemically opposed to the recognition of racial difference or specific culturally structured needs; rather, these two approaches must be in constant and iterative dialogue.

## Conclusions and directions for future research

We have drawn on an original dataset of 48 semi-structured interviews with REM students in two British HEIs to highlight their experiences with mental health help-seeking and, particularly, their understanding of the meaning and provision of “culturally appropriate” support. Our participants’ responses demonstrate the need for increased sensitivity in the way support is currently provided in their HEIs, and calls—beyond the circumstances of their own university environments—for a more general shift in thinking around how culturally appropriate support is expected to be provided by HEIs. Based on our dataset, we argue there is more than one way to provide culturally appropriate support and to focus on a single targeted approach. For some respondents, particularly British South Asian respondents, ethnic matching approaches could potentially even aggravate the problem.

As discussed, our dataset is not a random sample, and not designed to be generalizable. Nor does it seek to test the delivery of ethnic matching services in practice. Even the distinctions we can draw—such as the potential to see the negative aspects of ethnic matching in some respondents—must therefore be tentatively drawn, and it would be naïve on this basis to discredit the value of matching. Nonetheless, the adoption of culturally appropriate support mechanisms (and even more so, ethnic matching approaches) stand out from our student data as potentially problematic if they are constructed as isolated and protocol-driven interventions; equally, such approaches would not complement the multi-stranded nature of the “whole university approach” to which British HEIs claim they are otherwise committed. Viewing ethnic and/or culturally specific interventions as the sufficient or only solution to addressing the issues highlighted by our respondents places fundamental limits on engaging with how REM students understand mental health and help-seeking, and reduces their social and cultural beliefs, experiences, and preferences of their complexity. Above all, it overlooks the multiple iterative interactions between operative factors along the help-seeking process.

We recommend future research should build on the limitations of this study, and should consider exploring other intersectional factors that influence the relationship between REM students, their mental health and help-seeking attitudes and preferences. This research has specifically focused on race and ethnicity and its relationship to mental health help-seeking in the university environment as literature in this space remains relatively limited. Experiences and perceptions are complex; in order to develop counselling interventions and programmes that are truly culturally appropriate, person-centred, and tailored to REM students’ specific needs, the interactive effects of multiple facets of social identity should be consistently studied and identified. Overall, the implications of our findings are that HEIs, and their support services must be accountable and take responsibility for their previous and current failings, rather than prioritizing appearing “inclusive” or becoming “culturally appropriate”. Further, HEIs must clearly acknowledge that reducing mental health disparities between REM students and their White peers does not have an endpoint, and that a “tick-box” approach may well pull MHPs towards a zone of false confidence. Rather, it is essential to commit to an ongoing and reflexive process of embedding a person-centred approach within a framework recognizing cultural difference, and thus acknowledging the importance of humanity and trust-building when interacting with REM students. In this way, British HEIs may become mentally healthy environments where support services are accessible to and appropriate for all students.

## Supplementary Material

Supplemental Material

## Data Availability

To maintain participant anonymity, identifying data and study materials have been kept confidential and will not be made available to other researchers. The research has also not been preregistered. However, certain non-identifying participant characteristics demographics have been provided in Supplementary File 1

## Notes

1. UK-domiciled refers to students who were either born in the UK or have permanent residency i.e., non-international students.

2. They do appear to differ with regards to ethnic composition of their mental health professionals. In response to the author’s FOI requests [Reference: FOI.2021.06.356 and FOI.2021.286...], one declared that of the nine counselling and mental wellbeing staff employed at the HEI, five were from an REM background, though they did not disclose the specific ethnic group. Though vague, this response contrasted with their initial response to enquiry, which simply stated that counsellors were “a culturally and socially diverse team”. The other disclosed that they were “very diverse”, with practitioners that are well trained in issues of diversity and the impact of minority stress. Furthermore, if a student declared a preference for a specific practitioner, they could refer such students to an external counselling service who could provide counsellors of a specific ethnic origin or language.
