# Supplementary material for: Just ethnic matching? Racial and ethnic minority students and culturally appropriate mental health provision at British universities
Source: Int J Qual Stud Health Well-being. 2022 Sep 1;17(1):2117444. doi: 10.1080/17482631.2022.2117444 (PMC9448376; doi:10.1080/17482631.2022.2117444)
Supplement: Supplemental Material [file ZQHW_A_2117444_SM7822.docx]

Supplementary File 1

# **Just ethnic matching? Racial and ethnic minority students and mental health provision at British universities**

Table 1: Participant Characteristics

| **Characteristic** | **Number of Participants** |
| --- | --- |
| **University Attended** |  |
| University 1 | 28 |
| University 2 | 20 |
| Year of Study |  |
| Year 1 | 10 |
| Year 2 | 16 |
| Year 3 | 9 |
| Year 4 | 12 |
| Year 5 | 1 |
| **Participant Background** |  |
| Black African (BA) | 22 |
| Black Afro-Caribbean (BAC) | 10 |
| South Asian (SA) | 16 |
| Gender |  |
| Male | 18 |
| Female | 30 |
| **Religious Affiliation** |  |
| Christianity | 30 |
| Islam | 14 |
| Self-defined Spirituality | 3 |
| None | 1 |
| **Current (Professional) Mental (ill) Health Status** |  |
| Depression | 8 |
| Anxiety | 4 |
| Bipolar Disorder | 1 |
| Self -Defined | 21 |
| None | 14 |
| **Prior Help-Seeking Experience** |  |
| Professional Mental Health Services - External | 10 |
| Campus Support Services | 10 |
| Community or Faith-Based Services | 5 |
| None | 23 |
| **Current Help-Seeking Experience** |  |
| Professional Mental Health Services - External | 0 |
| Campus Support Services | 3 |
| Medication Only | 3 |
| Community/Faith-Based Services or Campus Chaplaincy | 0 |
| None | 42 |
| **Future Help-Seeking intentions** |  |
| Considering Professional Mental Health Services – On Campus | 0 |
| Considering Professional BAME-specific services – External | 7 |
| Considering faith-based services | 4 |
| Personal Strategies | 29 |
| None | 8 |
